# Intraspecific Variation of *Centruroides Edwardsii* Venom from Two Regions of Colombia

**DOI:** 10.3390/toxins6072082

**Published:** 2014-07-14

**Authors:** Sebastián Estrada-Gómez, Nelson Ivan Cupitra, Walter Murillo Arango, Leidy Johana Vargas Muñoz

**Affiliations:** 1 Facultad de Quimica Farmaceutica, Universidad de Antioquia UdeA, Carrera 53 No. 61-30, Medellín 050010, Colombia; 2 Programa de Ofidismo/Escorpionismo, Facultad de Química Farmacéutica, Universidad de Antioquia UdeA, Carrera 53 No. 61-30, Medellín 050010, Colombia; 3 Grupo de Investigación de Productos Naturales, Facultad de Ciencias, Universidad del Tolima, Barrio Santa Helena Parte Alta, Ibagué 731020, Colombia; E-Mails: ivancupitra@hotmail.com (N.I.C.); wmurillo@ut.edu.co (W.M.A.); 4 Facultad de Medicina, Universidad Cooperativa de Colombia, Calle 50 A No. 41-20, Medellín 050010, Colombia; E-Mail: johana2104@gmail.com

**Keywords:** intraspecific variation, *Centruroides edwardsii*, phospholipases, lethal activity, RP-HPLC

## Abstract

We report the first description studies, partial characterization, and intraspecific difference of *Centruroides edwardsii*, Gervais 1843, venom. *C. edwardsii* from two Colombian regions (Antioquia and Tolima) were evaluated. Both venoms showed hemolytic activity, possibly dependent of enzymatic active phospholipases, and neither coagulant nor proteolytic activities were observed. Venom electrophoretic profile showed significant differences between *C. edwardsii* venom from both regions. A high concentration of proteins with molecular masses between 31 kDa and 97.4 kDa, and an important concentration close or below 14.4 kDa were detected. RP-HPLC retention times between 38.2 min and 42.1 min, showed bands close to 14.4 kDa, which may correspond to phospholipases. RP-HPLC venom profile showed a well conserved region in both venoms between 7 and 17 min, after this, significant differences were detected. From Tolima region venom, 50 well-defined peaks were detected, while in the Antioquia region venom, 55 well-defined peaks were detected. Larvicidal activity was only detected in the *C. edwardsii* venom from Antioquia. No antimicrobial activity was observed using complete venom or RP-HPLC collected fractions of both venoms. Lethally activity (carried out on female albino swiss mice) was detected at doses over 19.2 mg/kg of crude venom. Toxic effects included distress, excitability, eye irritation and secretions, hyperventilation, ataxia, paralysis, and salivation.

## 1. Introduction

Over 400 million years of evolution, scorpions have developed a complex mixture of neurotoxins, enzymes, proteins, and antimicrobial and cytolytic peptides, most of these with a low molecular mass (below 10 kDa), designed to kill and paralyze prey, which also exhibit a wide variety of pharmacological and biochemical activities [1,2].

Venom from different species of *Centruroides* had been widely studied and characterized. Complete proteome from *C. noxius*, *C. sulfusus sulfusus*, and *C. sculpturatus* (now *C. exilicauda*) had been described showing different kinds of toxins, effecting different ionic channels like ERG K channel inhibitors, short chain peptides affecting Na^2+^ channels, among others [3,4,5,6,7]. As well, physiological characterization had been carried out from *C. sculpturatus* venom, showing specific inactivation of sodium channels permeability or inducing a transient shift in the activation voltage-dependence [8,9]. From *Centruroides tecomanus*, the proteome analysis and the cDNA transcriptome analysis had been reported [2], and from the venom of *Centruroides noxius*, *Centruroides limpidus limpidus*, and *Centruroides elegans*, different peptides with specific activity on Kv^+^ channels were isolated and identified [10,11,12]. Although no intraspecific differences had been reported in the *Centruroides* genus, from the Buthidae family, intraspecific differences of *Lychas mucronatus* from two regions of China have been reported [13]. Moreover, Abdel-Rahman *et al*. reported intraspecific variation in the Egyptian scorpion *Scorpio maurus palmatus* (Scorpionidae), which may be due to variation in the environmental conditions or more probably a reflection of the genetic diversity between populations [14].

Different peptides reported in scorpion venoms show an amphipathic α-helical structure, like those reported for different cationic antimicrobial molecules [15,16,17,18,19,20,21,22,23]. These peptides, show hemolytic, immune modulating, antibacterial, and insecticidal activities [1,23,24]. Antibacterial activity had been reported from different *Centruroides* species venom, such as *C. margaritatus*, active against *S. aureus*, *P. aeruginosa*, and *B. cereus* [25]. From the *Centruroides sulffusus sulffusus* venom, an antimicrobial peptide (AMP) named Css54, was isolated with a molecular mass of 2870.4 Da and a retention time of 53 min (in a RP-HPLC system), showing antimicrobial activity against *E. coli* and *S. aureus* [26]. Lethal activity had been described from *Centruroides exilicauda* and *C. sculpturatus* with a LD50 of 25 mg/kg and 3 mg/kg, respectively [27]. Insecticidal activity of the scorpions’ venoms had been reported mainly in the Buthidae family [28]. This activity is mediated largely by the great selectivity of peptides to ionic channels (sodium and calcium) and its amphipathic nature [28]. Symptoms, such as excitability, salivation, dyspnea, diarrhea, and temporary paralysis, had been reported in the venom of *Centruroides exilicauda* and *Centruroides sculpturatus* [27].

No studies have been found describing any characteristic of the *Centruroides edwardsii*, Gervais, 1843, venom. In Colombia, this is a wide spread distributed species in all the territory, especially in the Magdalena Valley region, including the Antioquia and Tolima provinces [29]. No reports, regarding this species venom, have been published until today, and we describe, for the first time, intraspecific differences and the biological characterizations (lethally, antimicrobial and larvicidal), biochemical (hemolytic, coagulant, and proteolytic), and the chromatographic and electrophoretic profile from this species.

## 2. Results

## 2.1. Venom Enzymatic Activity

**Figure 1:**
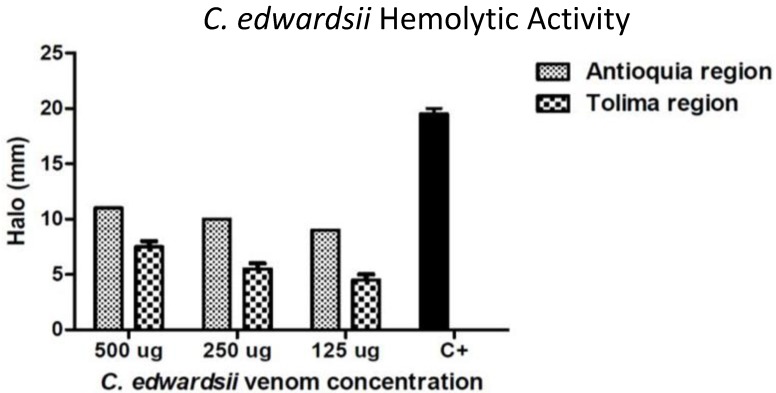
Indirect hemolytic activity, using calcium, of *C. edwardsii* venom from Antioquia and Tolima, at different concentrations (500, 250, and 125 µg). C+: Positive control, *Bothrops asper* crude venom (2 µg). Minimum hemolytic dose (MHeD) was defined as the amount of venom that induced a 20 mm diameter hemolytic halo. No deviations were detected in *C. edwardsii* venom from Antioquia.

*C. edwardsii* venom, from the Antioquia region caused an indirect hemolysis activity significantly different (*p* < 0.05) with respect to *C. edwardsii* venom from the Tolima region. *C. edwardsii* venom from Antioquia caused a MHeD of 2.21 mg, while *C. edwardsii* venom Tolima region, presented with a MHeD of 3.01 mg with calcium (Figure 1). Without calcium, no hemolytic activity was detected (data not shown). No coagulant or proteolytic activities were observed with these venoms (data not shown).

## 2.2. SDS-PAGE

**Figure 2:**
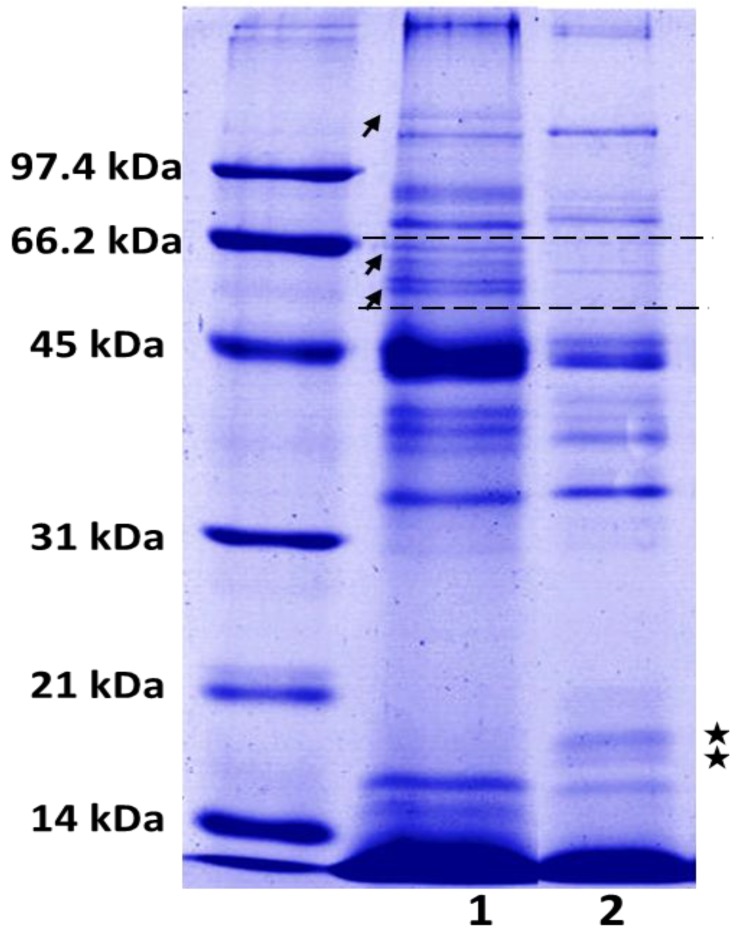
*C. edwardsii* crude venom (under reduced conditions) SDS-PAGE profile in a 12% gel from Antioquia (**1**) and Tolima (**2**). Venoms were loaded at concentrations of 1.5 µg/µL in final volume of 40 µL. Stars indicate proteins present in the Tolima *C. edwardsii* venom and absent in Antioquia *C. edwardsii* venom. Arrows indicate proteins absent Tolima *C. edwardsii* venom and present in Antioquia *C. edwardsii* venom.

Significant differences were showed between *C. edwardsii* venom from Antioquia and Tolima. Electrophoretic profile showed a high concentration of proteins with a molecular mass between 31 kDa and 97.4 kDa and important concentrations close or below 14.4 kDa (Figure 2). 

## 2.3. Reverse-Phase Chromatography

**Figure 3:**
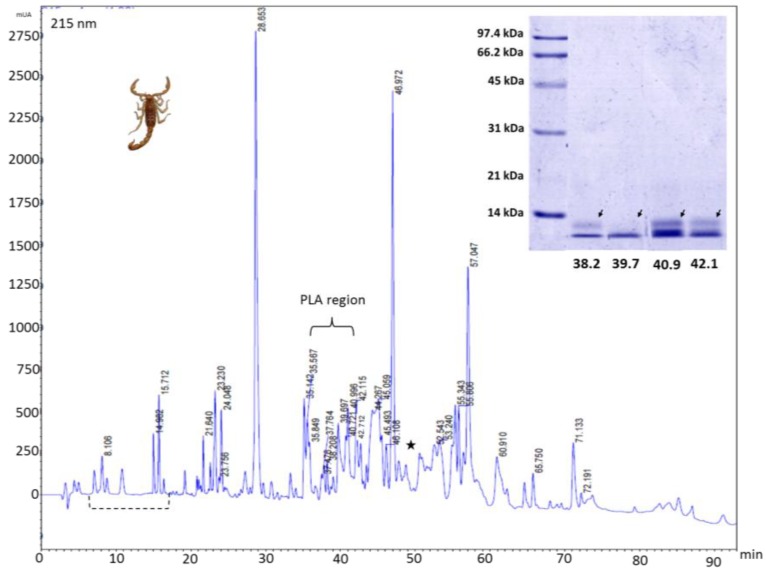
*C. edwardsii* venom (Tolima region, 0.5 mg) RP-HPLC chromatographic profile using a C18 column (250 mm × 4.6 mm). Elution gradient used: 0%–70% of acetonitrile (99% in TFA 0.1%). The run was monitored at 215 nm. Selected fractions were collected manually and analyzed by SDS-PAGE (insert) under reduced conditions. Insert arrows indicate possible phospholipases (PLA). 38.2, 39.7, 40.9, and 42.1 represents the elution time in the RP-HPLC system. Broken line indicates conserved region between both region venoms. Star indicates missing main peak compared with Antioquia venom at 49.1 min.

**Figure 4:**
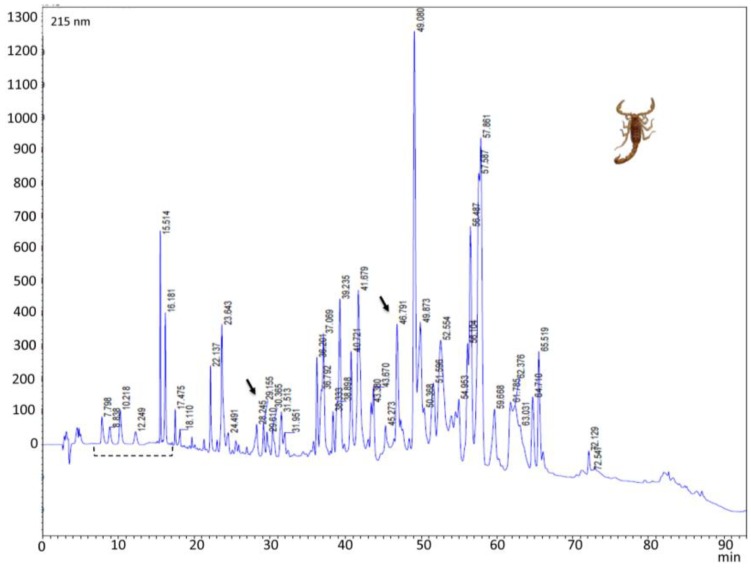
*C. edwardsii* venom (Antioquia region, 0.3 mg) RP-HPLC chromatographic profile using a C18 column (250 mm × 4.6 mm). Elution gradient used: 0%–70% of acetonitrile (99% in TFA 0.1%). The run was monitored at 215 nm. Broken line indicates conserved region between both region venoms. Arrows indicates missing peaks compared with *C. edwardsii* venom from Tolima at 28.6 and 46.9 min.

**Figure 5:**
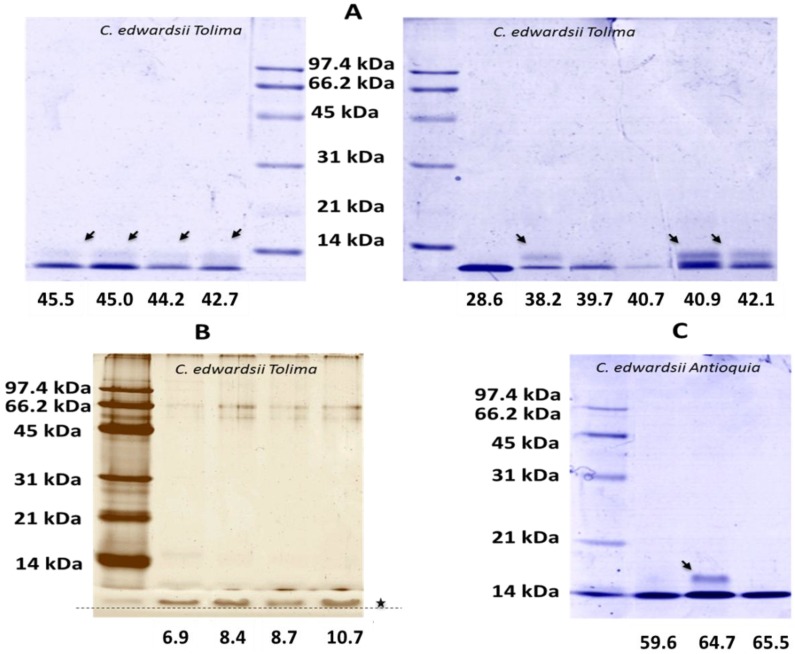
SDS-PAGE gel (12%) of RP-HPLC selected fractions from *C. edwardsii* venom under reduced conditions. (**A**) venom from the Tolima region, commasie blue stained; (**B**) venom from the Tolima region, silver stained, allowing to see the presence of low molecular mass components; (**C**) venom from the Antioquia region. In all cases arrows indicates molecular mass components close to 14.4 kDa while star indicate low molecular mass components below 14.4 kDa. Numbers indicate the retention time in the respective chromatogram.

RP-HPLC venom profile showed a well conserved region in both venoms eluting between 7 min and 17 min (5% and 15% of ACN respectively) (Figure 3 and Figure 4). After 17 min, significant differences were detected in both venoms. Main well defined peaks present in the Tolima *C. edwardsii* venom (28.6 and 46.9 min), eluting between 23% and 36% of ACN, were shown with much less intensity in the Antioquia region’s venom (Figure 3 and Figure 4). While Main peak from Antioquia venom, eluting at 49.1 min, is missing in Tolima’s venom. From Tolima’s venom, 50 well-defined peaks were detected with 8 eluting in the phospholipase region, while in the Antioquia’s venom, 55 well-defined peaks were detected with 5 eluting in the phospholipase region (Figure 3 and Figure 4). HPLC selected fractions, showed a high concentration of components with a molecular mass close to 14.4 kDa, eluting after 35 min in both venoms (Figure 2 and Figure 5). RP-HPLC retention times of 38.2, 39.7, 40.9, and 42.1 min, showed bands close to 14.4 kDa (Figure 3, insert), which may correspond to phospholipases. Silver stained gel allowed us to see low molecular mass components (Figure 5B). *C. edwardsii* venom from Antioquia showed similar characteristics as expressed above (data not shown), the only difference is the fraction with a retention time of 64.7 min, where a band over 14.4 kDa is observed (Figure 5C).

## 2.4. Toxicological and Biological Activities

*C. edwardsii* (from Tolima region) lethal activity indicated toxic effects at all tested dose (Table 1). Toxicological symptoms appeared after 5 min of venom injection. After 35 min, group 1 showed piloerection and diarrhea; group 2 presented this symptoms plus distress, excitability, eye irritation (including secretions), and hyperventilation; and group 3 showed all mentioned symptoms plus signs of pain, ataxia, paralysis, and salivation. After 2 h, individuals from group 1 presented eye irritation. No changes were observed in group 2 and 3, only one individual from group 3 was sacrificed (for ethical reasons) since the symptoms presented indicated extreme suffering. Lungs from this mouse were removed and generalized hemorrhage was observed respect negative control indicating a probably systemic hemorrhage (data not shown). All individuals recovered after 20 h. No antimicrobial activity was detected at the concentrations evaluated (data not shown) in both venoms. Only *C. edwardsii* from Antioquia, showed moderate insecticidal activity (Table 2).

**Table 1:** *C. edwardsii* venom toxic activity and comparsion with *C. sculpturatus* and *C. exilicauda*. Test was carried out on female albino swiss mice. PBS pH: 7.2 were used to dilute all doses and as negative control.

Venom dose (mg/kg)	*C. edwardsii*	*C. sculpturatus* [27]	*C. exilicauda* [27]
4.8	Toxic	Lethal	Non toxic
9.6	Toxic	Lethal	Non toxic
19.2	Toxic	Not tested	Toxic

**Table 2:** *C. edwardsii* venom insecticidal activity. Test was carried out on female albino swiss mice. PBS pH: 7.2 were used to dilute all doses and as negative control.

Venom concentration	Live larvae	Death larvae	Total	Mortality %
*C. edwardsii* from Antioquia				
400 µg	3	2	5	40
*C. edwardsii* from Tolima				
500 µg	5	0	5	0
250 µg	5	0	5	0
125 µg	5	0	5	0
62.5 µg	5	0	5	0

## 3. Discussion

We report the first biological and biochemical characterization of the *Centruroides edwardsii* venom, which is a species widely distributed in Colombia, among the Tolima and Antioquia regions, Magdalena River Valley [29].

*Centruroides* venom have been widely studied as a rich source of sodium and potassium channels modulators, and different antimicrobial peptides [26]. Although no venom characterization has been reported from this species, Colombian *C. edwardsii* venom agreed with the description of other *Centruroides* species like *C. tecomanus* [2] and different arachnids [30]. *C. edwardsii* venom exhibited an indirect hemolytic activity, which could be depend of phospholipases, since, when the assay was performed with calcium, the venom enhanced the activity, and without calcium no activity was performed (data not shown); furthermore, chromatograms (Figure 3 and Figure 4) show different peaks eluting between 37 min and 42 min (regular phospholipases eluting times in invertebrates) [1] with a molecular mass close to 14.4 kDa (Figure 3, insert). These characteristic properties have been reported in other *Centruroides* species (*C. tecomanus*), scorpions genus (*Opisthacanthus*), and arachnids (spider from the *Pamphobeteus* genus), were different phospholipases enzymes were identified with retention times between 37 min and 42 min and molecular masses between 13.7 kDa and 14.5 kDa [1,2,30]. A significant intraspecific difference was detected between both venoms, since MHeD was found to be higher in the Antioquia’s venom, probably due to higher concentration of phospholipases, since peaks eluting in the phospholipases region showed the same intensity with respect to the Tolima’s venom, but using half of the venom concentration. As expected with *Centruroides* venoms, no proteolytic or coagulant activity was observed, since no pro-coagulant or proteolytic proteins has been described in these venoms before [2,27,31], although some serine-proteases and metallo-proteases have been described in different Buthidae genus (*Buthus* and *Tityus*) [31,32,33]. In this genus, only linear cytolytic peptides or cationic antimicrobial peptides have been reported [2,26,31].

*C. edwardsii* crude venom SDS-PAGE shows a high concentration of protein content with a molecular mass between 31 kDa and 97.4 kDa, and an important concentration close or below 14.4 kDa (Figure 2). Clearly, a significant difference was detected between both venoms since the Tolima’s venom showed two bands between 14.4 kDa and 21 kDa missing in the Antioquia’s venom (see stars in Figure 2) and the same venom showed three missing bands present in the Antioquia’s venom over the 45 kDa and one over 97 kDa (Figure 2). These results are in concordance with those reported in other scorpions and arachnids, where aspects like sex, age, and geographical zone could affect toxin expression and venom composition [13,30,34,35,36]. HPLC collected fractions with retention times of 38.2, 39.7, 40.9, and 42.1 min, showed bands close to 14.4 kDa (Figure 3, insert), which may correspond to phospholipases as reported in other scorpions and arachnids [1,2,30]. RP-HPLC selected fractions SDS-PAGE from both venoms, showed a high concentration of components with a molecular mass close to 14.4 kDa, eluting after 35 min in both venoms (Figure 5A), which is in concordance with Valdez-Velazquez * et al.* (2013), who reported, from *C. tecomanus* venom, different components eluting between 27 and 53 min with a molecular mass close to 13.7 kDa, 10.8 kDa, 10.8 kDa, 15.8 kDa, and 15.9 kDa, all others components showed molecular masses between 0.259 and 13.7 kDa [2]. Different authors reported peaks eluting between 20 and 50 min, corresponding to low molecular mass compounds (<14 kDa), which could affect Na^2+^ and K^+^ ionic channels [10,11,12,27,37,38] or showing a phospholipase nature [1,2,30]. A mass fingerprint should be performed to confirm *C. edwardsii* venom molecular masses.

RP-HPLC venom profile showed a well-conserved region in both venoms between 7 and 17 min, after this, significant intraspecific differences were detected. The difference was remarkably important in the Antioquia’s venom, where two, dominant, well-defined peaks (28.6 and 46.9 min), detected in the Tolima’s venom, are clearly shown with much less intensity (Figure 4). As reported in *C. tecomanus*, where 60 clear peaks were reported in the chromatogram [2], *C. edwardsii* venom, from the Antioquia and Tolima regions, showed 55 and 50 well defined peaks detected respectively, with around 12% eluting in the phospholipase region. SDS-PAGE electrophoresis, from RP-HPLC collected fractions, showed that a high percentage of components from *C. edwardsii* has molecular masses below 14 kDa, as reported with *C. tecomanus*, 47% of the components have a molecular mass between 1000 and 5500 Da [2]. These low molecular mass components could be the responsible of the neurotoxic effects observed in the lethal activity since most of the *Centruroides* Kv^+^ modulators channel have molecular masses between 3731 Da and 4833 Da and Na_v_^+^ channels modulators channels have molecular masses in the range of the 7500 Da [10,11,12,27,37,38].

As expected in arachnid venoms, *C. edwardsii* venom displayed neurotoxic activities (characteristic of ionic channels modulations), which had been previously reported in other *Centruroides* genus, such as *C. exilicauda*, *C. sculpturatus*, and *C. noxius* [27,28]. All neurotoxic manifestations are in concordance with those reported from *C. exilicauda* by Valdez-Cruz *et al*., where lethal doses were reported over 25 mg/kg [27]. This activity is highly co-related with the presence of sodium channel modifier peptides (SCMP) reported in different species of *Centruroides* [27,37]. This SCMP are probably present in this venom since this peptide shows a molecular mass close to the 7 kDa and, as seen in Figure 3A, the individual RP-HPLC fractions showed a similar molecular mass; and SCMP has elution times (in a RP-HPLC system with conditions similar to the one that we used) between 30 and 50 min [27,37,38]. Figure 4 and Figure 5 show a high concentration of peptides eluting between these times. Although lethality was not possible to be established for *C. edwardsii* venom at the doses evaluated, this activity could be found over 19.2 mg/kg, and very close to it, since one individual from group 3 showed extreme suffering. This is in concordance with the lethal doses reported to *C. exilicauda* [27]. Unlike the Tolima venom, only *C. edwardsii* from Antioquia showed insecticide activity; this difference should be more deeply analyzed as the presence of components corresponding to low molecular mass compounds affecting ionic channels (which is the main mechanism of action for insecticide activity) is evident in both venoms. From *C. noxius* and *C. Sulffusus sulffusus*, different toxins had been isolated affecting sodium channels and exhibiting insecticide activity [28,39,40,41]. 

## 4. Experimental Section

## 4.1. Venom Extraction

*C. edwardsii* scorpions coming from the Tolima (15 individuals) and the Antioquia provinces (25 individuals), both males and females (West and North-West Andean regions, respectively), were kept in captivity in the Universidad del Tolima and Universidad de Antioquia Serpentarium, respectively, with water and food *ad libitum* in a polyphagic diet of invertebrates. Venom extraction process was carried out using electro-stimulation. Cupper electrodes, impregnated with water, were carefully positioned in the telson and electrical stimuli of 45 V were applied twice, with an interval of 5 s using a JRM electro-stimulator (model 06, series 007, Colombia). Collected venom was transferred to dry vials, lyophilized, and stored at −20 °C until use.

## 4.2. Indirect Hemolytic Activity

To establish any possible PLA_2_ activity, indirect hemolysis was performed in agarose erythrocyte-egg yolk gels, according to Gutiérrez * et al.* [42], using 0.8% agarose dissolved in PBS (0.12 M NaCl, 0.04 M sodium phosphate in distilled water), pH 7.2, and CaCl_2_ 0.1 M. An additional plate, without CaCl_2_ and egg yolk, was performed to verify that hemolytic activity is due to the PLA_2_ presence. Minimum hemolytic dose (MHeD) was defined as the amount of venom that induced a 20 mm diameter hemolytic halo. The experiments were performed in triplicate. As a positive control, 2 µg of *Bothrops asper* venom were used.

## 4.3. Coagulant Activity

Coagulant activity of venom was assessed on citrated human plasma. Samples of 100 μL of various concentrations of the venom were added to aliquots of 200 μL plasma, previously incubated at 37 °C. Clotting times were recorded in a HumaClot Junior coagulometer (Human; Wiesbaden, Germany), and the minimum coagulant doses (MCD) for plasma or fibrinogen were determined; the MCD corresponds to the amount of venom that induces clotting in 60 s [43].

## 4.4. Proteolytic Activity

Proteolytic activity was measured on azocasein (Sigma–Aldrich, St. Louis, MO, USA) according to Wang *et al*. [44] with some modifications. Briefly, 20, 10, 5, 2.5, and 1.25 μg of the venom were dissolved in 20 μL of 25 mm Tris (0.15 M NaCl, 5 mm CaCl2), pH 7.4, (in order to obtain 1.0, 0.5, 0.25, 0.125, and 0.0625 μg/μL). These solutions were incubated with 10 mg/mL of azocasein previously diluted in the same buffer. After 90 min of incubation at 37 °C, the reaction was stopped adding 200 μL of trichloroacetic acid. Samples were then centrifuged at 360 g for 5 min. Supernatant (100 μL) was mixed with an equal volume of 0.5 M NaOH, and the absorbance was measured at 450 nm. Results are shown as a unit of proteolytic activity, which corresponds to the amount of enzyme that induces a change in absorbance of 0.2.

## 4.5. Larvicidal Activity

The method established by the World Health Organization [45] was followed with a few modifications. Fifty microliters of *C. edwardsii* venom at different concentrations (500, 250, 125, and 62.5 µg) were added to a tube containing 450 µL of saline solution, 0.90%, and 5 *Aedes aegypti* larvae*.* A saline solution, 0.90%, was used as a negative control whereas a solution of piperazine was used as a positive control. The solutions were kept at room temperature 12 h in a light and dark photoperiod. The counting of death larvae (larvae with no movement) was performed on each tube at 24 h to 48 h. This procedure was performed in duplicate, with approval of the Universidad de Antioquia animal ethical committee.

## 4.6. Antimicrobial Activity

Antibiotic susceptibility tests were performed as proposed by Bauer * et al.* [46], and the Clinical and Laboratory Standards Institute (CLSI) [6], with some modifications. *Escherichia coli* (ATCC 25922) and *Staphylococcus aureus* (ATCC 25923) were grown on Mueller-Hinton agar (MH) and then suspended in 5 mL of MH sterile broth. Turbidity was measured at 600 nm and adjusted to 0.5 absorbance which corresponds to 1.5 × 10^−4^ colony forming units (CFU). Ten microliters of each venom dose (500 and 250 µg) and RP-HPLC collected fractions were added and incubated at 37 °C during 24 h. Physiology saline solution was used as negative control, and chloramphenicol (*Phyto* Technology Laboratories) was used as a reference control. Each test was performed in duplicate.

## 4.7. Electrophoretic Profile

*C. edwardsii* crude venoms electrophoretic profile and HPLC fractions (selected and collected), were analyzed using sodium dodecyl sulfate polyacrylamide gels (SDS-PAGE) according to Laemmli [47] on 12% gels, and stained with Coomassie blue R-250 and silver (Kit 161-0449, Bio-Rad). Molecular weights were estimated using standard markers (Bio-Rad).

## 4.8. Chromatographic Profile

One milligram of whole venom was dissolved in 200 μL of solution A (0.1% TFA in water) and centrifuged at 2300 g. The supernatant was then applied to a reverse-phase RESTEK C18 column (250 × 4.6 mm), and separated on a Shimadzu Prominence HPLC. Proteins were eluted by a gradient towards solution B (0.1% TFA in acetonitrile) as follows: 5% B for 5 min, 5%–15% B over 10 min, 15%–45% B over 60 min, and 45%–70% B over 12 min at a flow rate of 1.0 mL/min [48]. The chromatographic run was monitored at 215 nm, fractions were collected, lyophilized, and stored until use.

## 4.9. Toxicity

Toxicity test was carried out on female albino swiss mice of approximately 26 g body weight as described by Valdez-Cruz * et al.* [27]. Different amounts of crude venom from *C. edwardsii* were tested in parallel; 4.8 mg/kg, 9.6 mg/kg and 19.2 mg/kg (group 1, group 2 and group 3 respectively). Injections were performed intraperitoneally using PBS (phosphate buffered saline, containing 0.15 mm NaCl, 0.1 mm sodium phosphate at pH 7.4) as vehicle and negative control. The intoxication levels were called “non-toxic”, when the animals showed no symptoms of envenoming within 20 h after testing, or showed the same symptoms as the control mice injected with 100 μL of buffer alone (PBS). “Toxic” means that the mice showed symptoms such as: piloerection, excitability, salivation, lacrimation, dyspnea, diarrhea, and temporary paralysis, but recovered within 20 h. “Lethal” means that the mice showed some or all the symptoms of intoxication and died within 20 h after injection. Three mice were used in each dose and negative control with approval of the Universidad de Antioquia animal ethical committee.

## 4.10. Statistical Analysis

Results were expressed as mean ± standard error media (S.E.M.) and statistical comparisons were done using an ANOVA with a Bonferroni post-test assuming a significance of *p* < 0.05. All data analysis was done using GraphPad PRISM 5 (GraphPad Software, Inc., La Jolla, CA, USA).

## 5. Conclusions

In conclusion, *C. edwardsii* venom showed clearly intraspecific differences in the composition of the scorpion venom collected from both localities. This probably reflects innate individual variation in venom synthesis and expression. Qualitative variations in the venom composition of scorpions of the same species could partially explain the disparity of biochemical and biological assays. Local environmental conditions and geographical separation could play a major role in the intraspecific variation of *C. edwardsii* venom from Antioquia and Tolima.

## References

[1] Schwartz E.F., Camargos T.S., Zamudio F.Z., Silva L.P., Bloch C., Caixeta F., Schwartz C.A., Possani L.D. (2008). Mass spectrometry analysis, amino acid sequence and biological activity of venom components from the brazilian scorpion opisthacanthus cayaporum. Toxicon.

[2] Valdez-Velazquez L.L., Quintero-Hernandez V., Romero-Gutierrez M.T., Coronas F.I., Possani L.D. (2013). Mass fingerprinting of the venom and transcriptome of venom gland of scorpion centruroides tecomanus. PLoS One.

[3] Nastainczyk W., Meves H., Watt D.D. (2002). A short-chain peptide toxin isolated from centruroides sculpturatus scorpion venom inhibits ether-a-go-go-related gene k(+) channels. Toxicon.

[4] Gurrola G.B., Moreno-Hagelsieb G., Zamudio F.Z., Garcia M., Soberon X., Possani L.D. (1994). The disulfide bridges of toxin 2 from the scorpion centruroides noxius hoffmann and its three-dimensional structure calculated using the coordinates of variant 3 from centruroides sculpturatus. FEBS Lett..

[5] Gurrola G.B., Rosati B., Rocchetti M., Pimienta G., Zaza A., Arcangeli A., Olivotto M., Possani L.D., Wanke E. (1999). A toxin to nervous, cardiac, and endocrine erg k+ channels isolated from centruroides noxius scorpion venom. FASEB J..

[6] Clinical and Laboratory Standards Institute (2009). Performance Standards for Antimicrobial Susceptibility Testing, Nineteenth Informational Supplement.

[7] Watt D.D., Simard J.M. (1984). Neurotoxic proteins in scorpion venom. Toxin Rev..

[8] Meves H., Rubly N., Watt D.D. (1984). Voltage-dependent effect of a scorpion toxin on sodium current inactivation. Pflug. Archiv..

[9] Meves H., Simard J.M., Watt D.D. (1984). Biochemical and electrophysiological characteristics of toxins isolated from the venom of the scorpion centruroides sculpturatus. J. Phys..

[10] Coronas F.I., Balderas C., Lopez L.P., Possani L.D., Gurrola G.B. (2005). Amino acid sequence determination and chemical synthesis of cllerg1 (gamma-ktx1.5), a K^+^ channel blocker peptide isolated from the scorpion centruroides limpidus limpidus. J. Braz. Chem. Soc..

[11] Jouirou B., Mosbah A., Visan V., Grissmer S., M’Barek S., Fajloun Z., Van Rietschoten J., Devaux C., Rochat H., Lippens G. (2004). Cobatoxin 1 from centruroides noxius scorpion venom: Chemical synthesis, three-dimensional structure in solution, pharmacology and docking on K^+^ channels. Biochem. J..

[12] Olamendi-Portugal T., Somodi S., Fernandez J.A., Zamudio F.Z., Becerril B., Varga Z., Panyi G., Gaspar R., Possani L.D. (2005). Novel alpha-ktx peptides from the venom of the scorpion centruroides elegans selectively blockade kv1.3 over ikca1 k+ channels of t cells. Toxicon.

[13] Ruiming Z., Yibao M., Yawen H., Zhiyong D., Yingliang W., Zhijian C., Wenxin L. (2010). Comparative venom gland transcriptome analysis of the scorpion lychas mucronatus reveals intraspecific toxic gene diversity and new venomous components. BMC Genomics.

[14] Abdel-Rahman M.A., Omran M.A., Abdel-Nabi I.M., Ueda H., McVean A. (2009). Intraspecific variation in the egyptian scorpion scorpio maurus palmatus venom collected from different biotopes. Toxicon.

[15] Cao L.Y., Dai C., Li Z.J., Fan Z., Song Y., Wu Y.L., Cao Z.J., Li W.X. (2012). Antibacterial activity and mechanism of a scorpion venom peptide derivative *in vitro* and *in vivo*. PLoS One.

[16] Cao L.Y., Li Z.J., Zhang R.H., Wu Y.L., Li W.X., Cao Z.J. (2012). Stct2, a new antibacterial peptide characterized from the venom of the scorpion scorpiops tibetanus. Peptides.

[17] Cociancich S., Goyffon M., Bontems F., Bulet P., Bouet F., Menez A., Hoffmann J. (1993). Purification and characterization of a scorpion defensin, a 4kda antibacterial peptide presenting structural similarities with insect defensins and scorpion toxins. Biochem. Biophys. Res. Commun..

[18] Corzo G., Escoubas P., Villegas E., Barnham K.J., He W.L., Norton R.S., Nakajima T. (2001). Characterization of unique amphipathic antimicrobial peptides from venom of the scorpion pandinus imperator. Biochem. J..

[19] Corzo G., Villegas E., Gomez-Lagunas F., Possani L.D., Belokoneva O.S., Nakajima T. (2002). Oxyopinins, large amphipathic peptides isolated from the venom of the wolf spider oxyopes kitabensis with cytolytic properties and positive insecticidal cooperativity with spider neurotoxins. J. Biol. Chem..

[20] Diaz P., D’Suze G., Salazar V., Sevcik C., Shannon J.D., Sherman N.E., Fox J.W. (2009). Antibacterial activity of six novel peptides from tityus discrepans scorpion venom. A fluorescent probe study of microbial membrane Na+ permeability changes. Toxicon.

[21] Miyashita M., Sakai A., Matsushita N., Hanai Y., Nakagawa Y., Miyagawa H. (2010). A novel amphipathic linear peptide with both insect toxicity and antimicrobial activity from the venom of the scorpion isometrus maculatus. Biosci. Biotechnol. Biochem..

[22] Ramirez-Carreto S., Quintero-Hernandez V., Jimenez-Vargas J.M., Corzo G., Possani L.D., Becerril B., Ortiz E. (2012). Gene cloning and functional characterization of four novel antimicrobial-like peptides from scorpions of the family vaejovidae. Peptides.

[23] Zeng X.C., Corzo G., Hahin R. (2005). Scorpion venom peptides without disulfide bridges. IUBMB Life.

[24] Giangaspero A., Sandri L., Tossi A. (2001). Amphipathic alpha helical antimicrobial peptides. Eur. J. Biochem..

[25] Escobar E., Flores L., Rivera C. (2008). Antibacterial peptides from hadruroides mauryi and centruroides margaritatus venom. Rev. Peru. Boil..

[26] Garcia F., Villegas E., Espino-Solis G.P., Rodriguez A., Paniagua-Solis J.F., Sandoval-Lopez G., Possani L.D., Corzo G. (2013). Antimicrobial peptides from arachnid venoms and their microbicidal activity in the presence of commercial antibiotics. J. Antibiot..

[27] Valdez-Cruz N.A., Davila S., Licea A., Corona M., Zamudio F.Z., Garcia-Valdes J., Boyer L., Possani L.D. (2004). Biochemical, genetic and physiological characterization of venom components from two species of scorpions: Centruroides exilicauda wood and centruroides sculpturatus ewing. Biochimie.

[28] Gurevitz M., Karbat I., Cohen L., Ilan N., Kahn R., Turkov M., Stankiewicz M., Stühmer W., Dong K., Gordon D. (2007). The insecticidal potential of scorpion β-toxins. Toxicon.

[29] De Armas L.F., Sarmiento D.L., Florez E. (2012). Composición del género centruroides marx, 1890 (scorpiones:Buthidae) en colombia, con al descripción de una nueva especie. Boletínde la Sociedad Entomológica Aragonesa.

[30] Estrada-Gomez S., Munoz L.J.V., Castillo J.C.Q. (2013). Extraction and partial characterization of venom from the colombian spider pamphobeteus aff. Nigricolor (aranae:Theraphosidae). Toxicon.

[31] De la Vega R.C.R., Schwartz E.F., Possani L.D. (2010). Mining on scorpion venom biodiversity. Toxicon.

[32] Fletcher P.L., Fletcher M.D., Weninger K., Anderson T.E., Martin B.M. (2010). Vesicle-associated membrane protein (vamp) cleavage by a new metalloprotease from the brazilian scorpion tityus serrulatus. J. Biol. Chem..

[33] Soudani N., Gharbi-Chihi J., Srairi-Abid N., Yazidi C.M., Planells R., Margotat A., Torresani J., El Ayeb M. (2005). Isolation and molecular characterization of lvp1 lipolysis activating peptide from scorpion buthus occitanus tunetanus. Biochim. Biophys. Acta.

[34] Binford G.J. (2001). An analysis of geographic and intersexual chemical variation in venoms of the spider tegenaria agrestis (agelenidae). Toxicon.

[35] De Sousa L., Borges A., Vasquez-Suarez A., Op den Camp H.J., Chadee-Burgos R.I., Romero-Bellorin M., Espinoza J., de Sousa-Insana L., Pino-Garcia O. (2010). Differences in venom toxicity and antigenicity between females and males tityus nororientalis (buthidae) scorpions. J. Venom Res..

[36] Herzig V., Hodgson W.C. (2009). Intersexual variations in the pharmacological properties of coremiocnemis tropix (araneae, theraphosidae) spider venom. Toxicon.

[37] Espino-Solis G.P., Estrada G., Olamendi-Portugal T., Villegas E., Zamudio F., Cestele S., Possani L.D., Corzo G. (2011). Isolation and molecular cloning of beta-neurotoxins from the venom of the scorpion centruroides suffusus suffusus. Toxicon.

[38] Saucedo A.L., del Rio-Portilla F., Picco C., Estrada G., Prestipino G., Possani L.D., Delepierre M., Corzo G. (2012). Solution structure of native and recombinant expressed toxin cssii from the venom of the scorpion centruroides suffusus suffusus, and their effects on nav1.5 sodium channels. Biochim. Biophys. Acta.

[39] Martin M.F., Garcia y Perez L.G., El Ayeb M., Kopeyan C., Bechis G., Jover E., Rochat H. (1987). Purification and chemical and biological characterizations of seven toxins from the mexican scorpion, centruroides suffusus suffusus. J. Biol. Chem..

[40] Pintar A., Possani L.D., Delepierre M. (1999). Solution structure of toxin 2 from centruroides noxius hoffmann, a beta-scorpion neurotoxin acting on sodium channels. J. Mol. Biol..

[41] Vazquez A., Tapia J.V., Eliason W.K., Martin B.M., Lebreton F., Delepierre M., Possani L.D., Becerril B. (1995). Cloning and characterization of the cdnas encoding Na+ channel-specific toxins 1 and 2 of the scorpion centruroides noxius hoffmann. Toxicon.

[42] Gutierrez J.M., Avila C., Rojas E., Cerdas L. (1988). An alternative in vitro method for testing the potency of the polyvalent antivenom produced in costa rica. Toxicon.

[43] Theakston R.D., Reid H.A. (1983). Development of simple standard assay procedures for the characterization of snake venom. Bull. World Health Organ..

[44] Wang W.J., Shih C.H., Huang T.F. (2004). A novel p-i class metalloproteinase with broad substrate-cleaving activity, agkislysin, from agkistrodon acutus venom. Biochem. Biophys. Res. Commun..

[45] World Health Organization Guidelines for laboratory and field testing of mosquito larvicides. http://whqlibdoc.who.int/hq/2005/WHO_CDS_WHOPES_GCDPP_2005.13.pdf?ua=1.

[46] Bauer A.W., Kirby W.M., Sherris J.C., Turck M. (1966). Antibiotic susceptibility testing by a standardized single disk method. Am. J. Clin. Pathol..

[47] Laemmli U.K. (1970). Cleavage of structural proteins during the assembly of the head of bacteriophage t4. Nature.

[48] Fernandez J., Gutierrez J.M., Angulo Y., Sanz L., Juarez P., Calvete J.J., Lomonte B. (2010). Isolation of an acidic phospholipase a2 from the venom of the snake bothrops asper of costa rica: Biochemical and toxicological characterization. Biochimie.

